# Potential therapies for non-coding RNAs in breast cancer

**DOI:** 10.3389/fonc.2024.1452666

**Published:** 2024-09-20

**Authors:** Ruonan Li, Yuxin Ji, Ruyin Ye, Guohui Tang, Wenrui Wang, Changjie Chen, Qingling Yang

**Affiliations:** ^1^ Anhui Provincial Key Laboratory of Tumor Evolution and Intelligent Diagnosis and Treatment, Bengbu Medical University, Bengbu, Anhui, China; ^2^ School of Laboratory Medicine, Bengbu Medical University, Bengbu, Anhui, China; ^3^ Department of Life Sciences, Bengbu Medical University, Bengbu, Anhui, China; ^4^ Institute of Health and Medicine, Hefei Comprehensive National Science Center, Hefei, Anhui, China

**Keywords:** non-coding RNA, breast cancer, ncRNA therapy, long non-coding RNA, RNA interference

## Abstract

Breast cancer (BC) is one of the frequent tumors that seriously endanger the physical and mental well-being in women with strong heterogeneity, and its pathogenesis involves multiple risk factors. Depending on the type of BC, hormonal therapy, targeted therapy, and immunotherapy are the current systemic treatment options along with conventional chemotherapy. Despite significant progress in understanding BC pathogenesis and therapeutic options, there is still a need to identify new therapeutic targets and develop more effective treatments. According to recent sequencing and profiling studies, non-coding (nc) RNAs genes are deregulated in human cancers via deletion, amplification, abnormal epigenetic, or transcriptional regulation, and similarly, the expression of many ncRNAs is altered in breast cancer cell lines and tissues. The ability of single ncRNAs to regulate the expression of multiple downstream gene targets and related pathways provides a theoretical basis for studying them for cancer therapeutic drug development and targeted delivery. Therefore, it is far-reaching to explore the role of ncRNAs in tumor development and their potential as therapeutic targets. Here, our review outlines the potential of two major ncRNAs, long non-coding RNAs (lncRNAs) and microRNAs (miRNAs) as diagnostic and prognostic biomarkers as well as targets for new therapeutic strategies in breast cancer.

## 1 Introduction

Human Genome Project information shows that > 90% of the genome is transcribed, however, only about 2% of the genome is translated, and non-coding (nc) RNAs make up 98% of the total RNAs. Compared to protein-coding genes, a growing number of studies have shown that ncRNAs play key roles in a variety of biological processes such as transcription, post-transcriptional modification, chromatin remodeling and signal transduction (1). Abnormal expression levels of many genes involved in breast cancer (BC) development are presumably influenced by ncRNA activity (2). In addition, ncRNAs can be therapeutically targeted and the delivery of ncRNAs can be based on the existing basis of RNAi and oligonucleotide delivery for targeting protein-coding mRNAs (3, 4). Therefore, understanding specific ncRNA signatures can help to understand the complex BC cellular mechanisms and facilitate research advances in the diagnosis and treatment of BC subtypes.

The two main classes of ncRNAs are the well-studied short microRNAs (miRNAs) and long non-coding RNAs (lncRNAs). LncRNA usually has more than 200 nucleotides, while miRNA has 19-28 nucleotides (5). Small RNA species in the usual sense, including miRNAs, piwi-interacting RNAs (piRNAs), and small interfering RNAs (siRNAs), which interact with Argonaute proteins (Ago proteins) to mediate RNA silencing effects (6). Whereas lncRNAs positively or negatively regulate BC cell multiplication, invasion, metastasis and stemness properties by regulating the expression of miRNAs or transcription factors (7). Dysregulation of both types of transcripts has been associated with every cancer studied to date and affects all major cancer hallmarks. A variety of RNA-based therapeutics have been developed, including antisense oligonucleotides (ASOs), small interfering RNAs (siRNAs), short hairpin RNAs (shRNAs), ASO-anti-microRNAs (antimiRs), miRNA mimics, miRNA sponges, therapeutic circular RNAs (circRNAs), and CRISPR-Cas9-based gene editing, and there are several excellent reviews describing these drugs (8, 9). In breast cancer therapy, ncRNAs can be used as potential therapeutic targets by designing specific siRNAs or miRNAs to inhibit tumor-promoting ncRNAs or by overexpressing certain tumor-suppressing mRNAs to treat breast cancer. The application of nanodrug delivery systems in breast cancer therapy has also shown great potential, for which promising nanodelivery/nanoparticle-based approaches have been developed using multiple molecules for systemic drug delivery and improved targeted delivery of tumor ncRNAs with reduced side effects. Despite the promise of ncRNA therapeutics, challenges such as complexity and diversity, stability issues, delivery systems, specificity, and more in-depth research need to be overcome to achieve clinically applicable applications. One of the greatest challenges in the field today is to illuminate the multiple functions and mechanisms of action of ncRNA, which is critical for determining its clinical relevance and developing its potential use as a biomarker or therapeutic target (10).

## 2 lncRNA

### 2.1 Biological functions of lncRNAs

LncRNA is a heterogeneous set of non-protein-coding transcripts greater than 200 nucleotides in length (11). Similar in biogenesis to mRNAs, lncRNAs are transcribed by RNA polymerase II and have conserved secondary structures, many of which are spliced, capped, and polyadenylated. The complexity of these transcripts arises from their multifaceted 3D structures, which change rapidly and give them the ability to perform multiple functions (12). Depending on the relative position of lncRNAs to protein-coding genes in the genome, they can be categorized as positive lncRNAs, antisense lncRNAs, bidirectional lncRNAs, intronic lncRNAs, intergenic lncRNAs, and enhancer lncRNAs (13). LncRNAs have been found to be involved in a variety of physiological and pathological cellular activities, such as adipogenesis, inflammation, cellular differentiation, and tumorigenesis, by interacting with chromatin, proteins, and RNAs in the nucleus or cytoplasm, and through genomic expression regulation in cis or trans, epigenetic modification, and post-transcriptional modulation (14–16). In the nucleus, lncRNAs can modify gene expression by interacting directly with DNA or chromatin regulators such as transcription factors and RNA-binding proteins, acting as enhancers, decoys, scaffolds, or guides. In the cytoplasm, lncRNAs decay mRNAs, regulate mRNA stability or translation, compete with miRNAs for binding mRNAs, and can be processed into miRNAs (17). Some of the potential therapeutic targets of lncRNAs are summarized in **Table 1**. Growing evidence suggests that lncRNAs play an important role in a variety of cellular processes such as proliferation, apoptosis, treatment resistance and metastasis in human cancers (18–20).

**Table 1:** Potential therapeutic targets of lncRNAs in breast cancer.

lncRNA	Cell	Target	Function	Reference
TYMSOS	MCF-7、MDA-MB-231	High expression upregulates CBX3 and promotes CBX3-mediated repression of ULBP3 transcription	Inhibits NK cell cytotoxicity and promotes metastasis and immune escape	(104)
NKILA	MUC1+breast cancer tissues	High expression downregulates NF-κB	Contributes to the AICD sensitivity of tumor-antigen-activated CTLs	(105)
GATA3-AS1	TNBC	High expression and up-regulation of COPS5 promotes PD-L1 deubiquitination	Promotes immune escape of TNBC cells	(106)
TINCR	UACC812、 MDA-MB-231	Acts as a molecular sponge for miR-199a-5p and upregulates the stability of USP20 mRNA, thereby promoting PD-L1 expression by inhibiting its ubiquitination	Induction of immune escape and promotion of disease progression in breast cancer	(107)
SNHG16	MCF-7、T-47D、MDA-MB-231、MDA-MB-468	Acts as a molecular sponge for miR-16-5p and enhances the TGF-β1/SMAD5 pathway to upregulate CD73 levels	Enhancement of effective immunosuppression by CD73+Vδ1 T cells	(108)
lncRNA Xist	MCF-7	Competes as a molecular sponge for miR-101 to regulate C/EBPα and KLF6	Promotion of Xist Expression in M1 Macrophages and Inhibition of miR-101 Expression in M2 Macrophages Inhibit Mammary Proliferation and Migratory Capacity	(109)
lncRNA-p21	4T1	High expression of targeted p53 eliminated MDM2 degradation to p53	Knockdown reverses the phenotype of TAMs and produces TNF-α to kill tumor cells, thus exerting anti-tumor functions	(110)
MALAT1	SK-BR-3	Targeting miR-485-3p to downregulate P-gp and Bcl-2 and upregulate Bax	Oncogenic and tumor-suppressive roles	(111)

### 2.2 lncRNA therapy in breast cancer

#### 2.2.1 Targeted therapies

Programmed cell death ligand 1 (PD-L1), an immune checkpoint protein frequently expressed in human cancers, promotes immune escape by binding to PD-1 on activated T cells (21). Some lncRNAs act specifically in cancer cells by regulating antigen presentation or PD-L1 expression. Lin et al. (22) found that lncRNA, a HIF-1α inhibitor at the translation level (HITT), coordinated with the regulator of G protein signal transduction 2 (RGS2), binds to the 5 ‘UTR of PD-L1 under IFN-γ stimulation, resulting in reduced PD-L1 translation. In human breast cancer, HITT/RGS2 was negatively correlated with PD-L1 expression, suggesting that HITT may suppress PD-L1 expression *in vivo*. The metastasis-associated lung adenocarcinoma transcript (Malat1) is an abundant lncRNA, and many studies have shown that its expression is upregulated in a variety of cancers (23–26). Using the TNBC cell line MDA-MB-231, Samir and colleagues demonstrated that miR-182-5p, in additon to down-regulating the expression of the tumor suppressor gene XIST in the same cells, can act as an oncomiR by promoting the up-regulation of oncogenic PD-L1 and the lncRNA MALAT1 (27). In addition to immunotherapy targeting immune checkpoint molecules, in a study of TNBC, Adewunmi et al. (28) found that Malat1 inhibition resulted in delayed primary tumor growth in macrophage-rich T12 tumor subtypes and neutrophil-rich 2208 L tumors and a significant reduction in tumor volume in both models after 14 days of treatment. By using Malat1 ASO, they found that they were able to knock down Malat1 RNA expression, which delayed primary tumor growth, decreased proliferation, and increased apoptosis. Also in a breast cancer model, Kumar et al. (29) found that deletion of Malat1 activates T cells and kills early metastatic cells, and its absence inhibits metastatic reactivation and restores dormancy. The use of Malat1 Gapmer locked nucleic acid (LNA) ASOs sufficiently inhibited the expression of Malat1 and Serpinb6b, significantly reducing lung colonization. This effect was associated with increased CD4+ and CD8+ T cell infiltration in micrometastases and decreased recruitment of Ly6G+ neutrophils. Therefore, targeting Malat1 may be a potential therapeutic avenue for the treatment of metastatic breast cancer.

**Figure 1:**
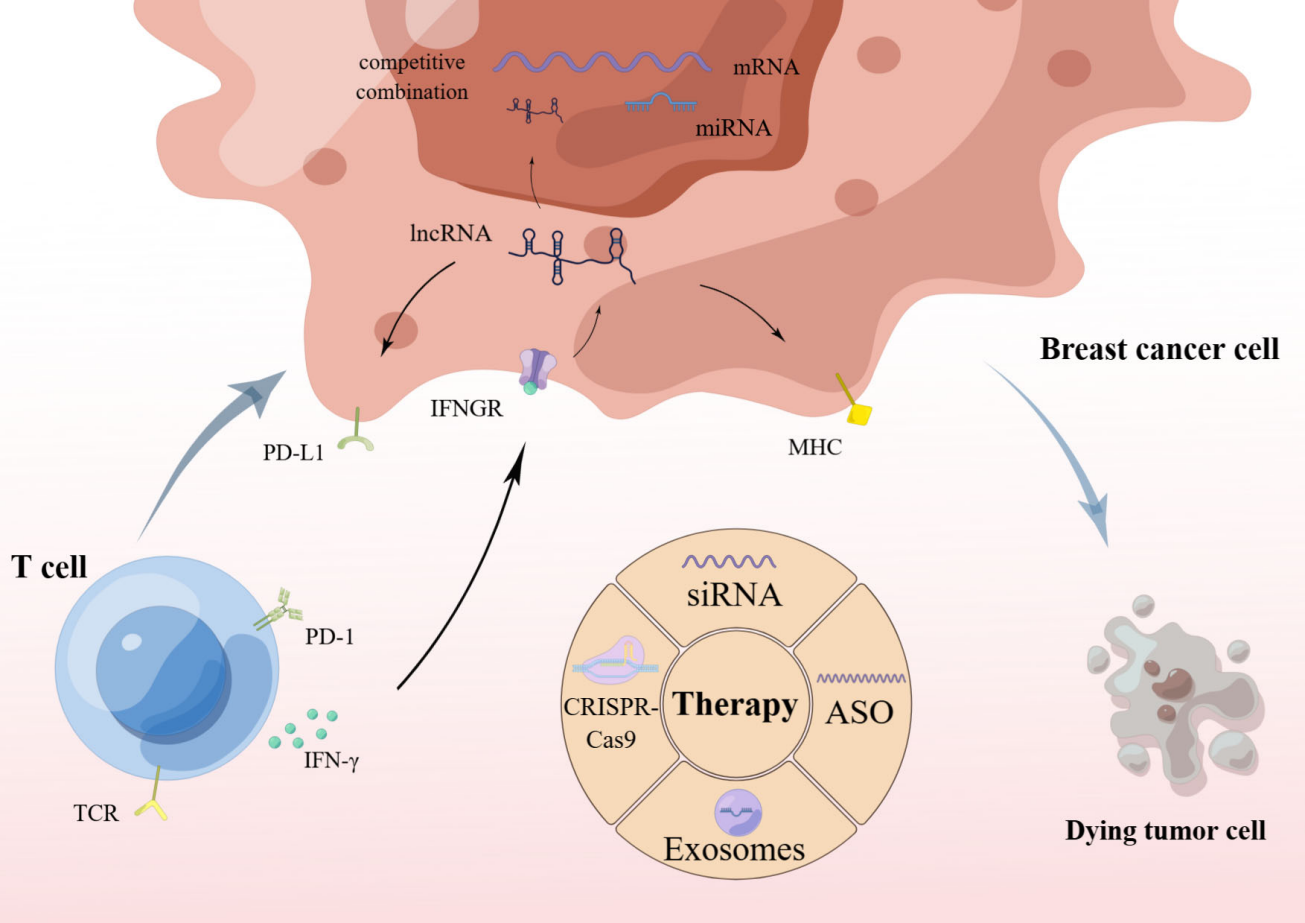
lncRNA functions and therapies.

Interferons have recently returned to the forefront of tumor biology research. Tumor cell response to conventional therapy is regulated by activation of the IFN pathway. In addition to cytotoxic drugs, blocking growth signaling pathways (such as EGFR and HER2 pathways) relies on IFN signal transduction. Zhang et al. (30) showed that blockade of IFN receptor 1 (IFNAR1) impaired the therapeutic effect of anti-HER2 monoclonal antibodies. LINC00624 promotes ADAR1 (adenosine deaminase RNA specific 1) RNA editing ability by binding to ADAR1. Expression of ADAR1, an A-to-I RNA editing protein that inhibits innate immune responses and is associated with the regulation of type I IFN responses, further inhibits IFN-induced expression of IFN-stimulated genes (ISGs), which stimulate antigen-presentation pathways, thereby recruiting immune cells and facilitating antiviral responses. Through mouse experiments, they found that xenograft tumors treated with ASO exhibited reduced ADAR expression. In addition, the expression levels of ISGs and innate immune response genes were significantly enhanced in ASO-treated xenograft tumors. Therefore, targeting LINC00624 by ASO significantly inhibited tumor cell proliferation, suppressed ADAR1 activity and promoted type I IFN response. In addition to, a study (31) found that IFN induced the expression of cytoplasmic lncRNA IFN-responsive nuclear factor-κB activator (IRENA) in macrophages, which triggered nuclear factor-κB signaling through dimerization of protein kinase R, followed by an increase in the production of pro-tumor inflammatory cytokines. The specificity of IRENA lncRNA expression in TAMs and its induction under chemotherapy make it a promising therapeutic site for avoiding chemoresistance and inhibiting cancer progression. The immune function of cancer-promoting and cancer-suppressing lncRNAs suggests that lncRNAs can be involved in regulating the crosstalk between tumors and immune cells during cancer onset and progression (**Figure 1**).

#### 2.2.2 lncRNA therapy in breast cancer chemoresistance

Development of therapeutic resistance and metastasis as major issues in breast cancer treatment (32). LncRNAs are dysregulated in various malignancies and interact with multiple RNAs and proteins to influence drug resistance. LncRNA DIO3OS has been found to be upregulated in breast cancer patients treated with aromatase inhibitors (AI). The mechanism of action of DIO3OS includes its interaction with polypyrimidine bundle-binding protein 1 (PTBP1), which stabilizes mRNA for lactate dehydrogenase A (LDHA), thereby upregulating LDHA expression and promoting glycolytic metabolism. That is, DIO3OS enhances aerobic glycolysis by regulating the splicing switch, thereby conferring a growth advantage to AI-resistant cells. Therefore, inhibition of LDHA activity by exploring DIO3OS knockdown approaches could re-sensitize breast tumor cells to anti-HER2 therapies (trastuzumab) or chemotherapies (paclitaxel) as a breast cancer treatment target (33).

In a study for the treatment of paclitaxel-resistant breast cancer, it was observed that LINC00115 was strongly upregulated in paclitaxel-resistant BCSC, and that LINC00115 acted as an RNA linker recruiting the SETDB1/PLK3 complex to activate the HIF1α signaling pathway (34). SETDB1 is an oncogene in breast cancer and play an important role in the treatment of endocrine therapy resistance (35, 36). Methylation of PLK3 leads to failure of HIF1α phosphorylation, which promotes HIF1α protein stability by inhibiting its ubiquitinated degradation pathway. HIF1 can enhance the stability of LINC00115 in turn, and this feedback loop further enhances BCSC characteristics, thereby promoting chemotherapy resistance and metastasis in breast cancer. Thus, inhibition of LINC00115 in combination with SETDB1 inhibitors significantly improved the efficiency of paclitaxel chemotherapy in an animal xenograft model of breast cancer metastasis.

Chen et al. (37) found that LINC02568 regulates estrogen/estrogen receptor-induced transcriptional activation of target genes in the cytoplasm by competitively binding miR-1233-5p to the estrogen receptor ESR1 mRNA itself, thereby trans-regulating the stability of ESR1 mRNA. LINC02568 is involved in transcriptional activation of neighboring genes CA12 by cis regulation in the nucleus, thereby participating in the maintenance of specific pH inside and outside tumor cells. LINC02568 is involved in the transcriptional activation of the neighboring gene CA12 in the nucleus through cis-regulation, which in turn is involved in the maintenance of specific pH inside and outside the tumor cell. ASO targeting LINC02568 significantly inhibited the growth and tumor formation of estrogen receptor-positive breast cancer cells and restored the sensitivity of tamoxifen-resistant breast cancer cells to tamoxifen. Therefore, the combination of ASO targeting LINC02568 and endocrine drugs or CA12 inhibitors has a synergistic effect on tumor growth inhibition.

LINC00460 was observed to be significantly elevated in doxorubicin-resistant breast cancer cells, and LINC00460, together with FUS, promotes MYC expression by influencing the efficiency of intron removal during mRNA maturation. Conversely, LINC00460 transcription is directly activated by c-MYC and forms a positive feedback loop in breast cancer cells, driving resistance to tamoxifen. The simultaneous depletion of LINC00460 and c-MYC inhibition remarkably re-sensitized ADR cells to Doxorubicin. In this context, Yang et al. (38) further suggested simultaneous antagonism of LINC00460 and c-MYC, which presumably efficiently abrogated the positive feedback loop and may represent a promising novel approach to improve therapeutic outcomes for patients with acquired resistance to Doxorubicin therapies. In another study of adriamycin resistance in breast cancer, Liu et al. (39) found that lncRNA aspartate-trna synthetase-antisense RNA 1 (DARS-AS1) was overexpressed in TNBC, and its silence effectively inhibited tumor growth and liver metastasis. They constructed a TNBC-specific natural nanomedicine delivery system, EXOs-CL4, which was loaded with DARS-AS1 siRNA and DOX (DARS-AS1 siRNA/DOX@EXOs-CL4) that synergistically inhibited tumor growth, metastasis, and anti-apoptotic effects (40). Resistance-causing lncRNAs can be used to develop new targeted and tailored therapies, providing a new approach to introducing promising personalized treatment modalities to overcome chemotherapy resistance in breast cancer patients.

## 3 miRNA

### 3.1 Biological functions of miRNAs

**Figure 2:**
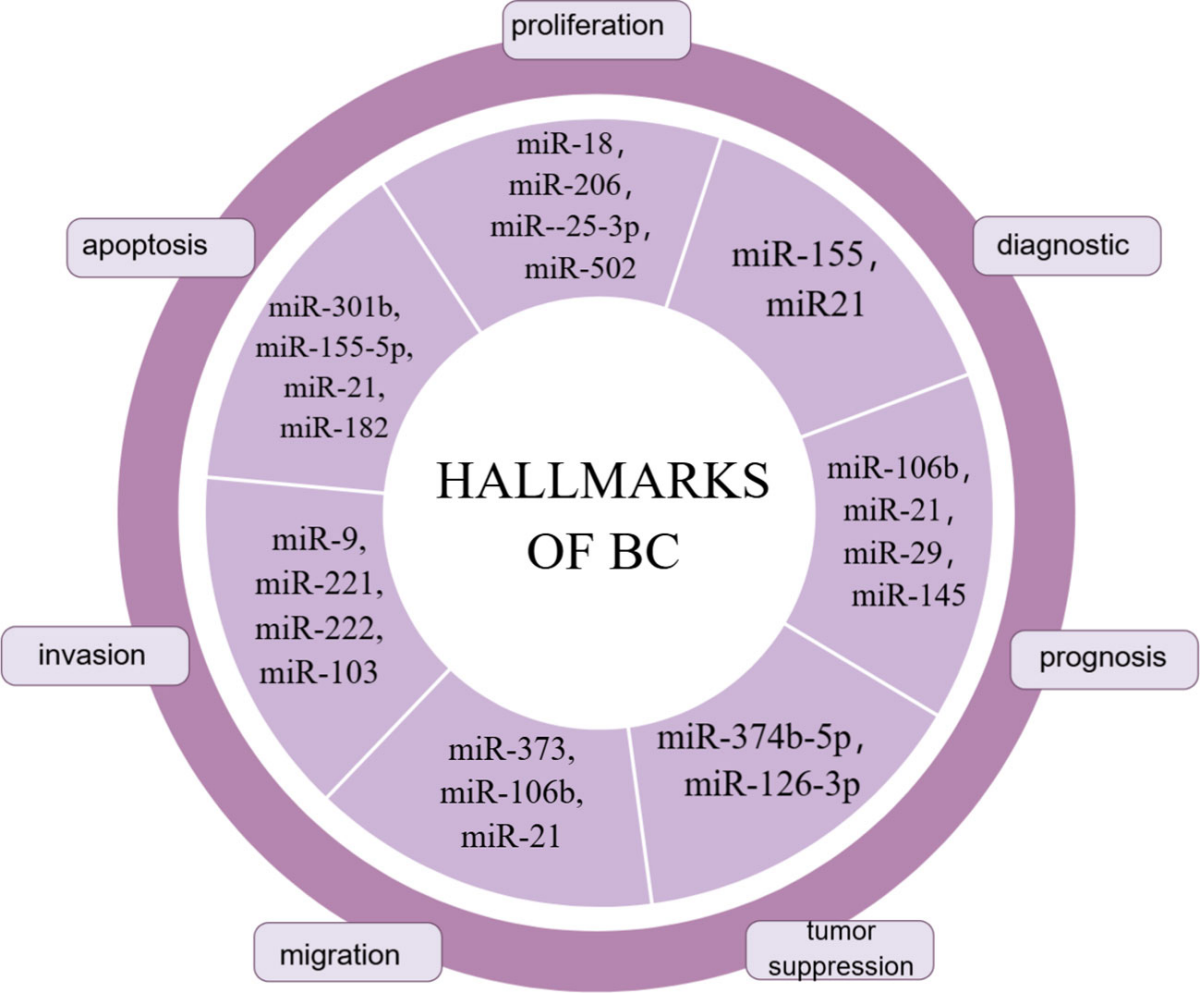
miRNA markers in breast cancer.

MiRNAs were the first to be discovered and analyzed in cancer (41, 42). MiRNA biogenesis is a multistep process: first, miRNAs are transcribed into pri-miRNAs by RNA polymerase II; second, pre-miRNAs are exported into the cytoplasm via exportin 5 (XPO5) after processing by the nuclear ribonuclease Drosha complex and DGCR8; Third, mature double-stranded miRNAs are generated and loaded into the rna-induced silencing complex (RISC) by processing mediated by the RNase III enzyme Dicer and TAR RNA binding protein 2 (TARBP2) (43–45). MiRNA gene expression usually occurs post-transcriptionally, an effect known as gene silencing, which is established primarily through mRNA cutting, translational repression, or DNA methylation. These molecules participate in the post-transcriptional repression of specific gene expression by binding to the 3’ untranslated region of the target mRNA, a process that requires the miRNA to bind to Ago proteins, which are the core components of RISC. Once loaded onto Ago proteins, miRNAs can direct RISCs to reach complementary target mRNAs for translational repression or mRNA degradation (46). However, this mechanism is not exclusive; binding of miRNAs to the 5’-UTR is also possible and induces activation or repression of translation (47, 48). MiRNAs play a crucial role in regulating transcription and post-transcriptional gene expression by specifically interacting with target mRNAs. Moreover, miRNAs can target multiple genes through a single pathway. For example, the miR-15-miR-16 cluster down-regulates several anti-apoptotic factors including BCL-2 and MCL1 (49). Thus, therapies using miRNAs or targeting miRNAs also have the potential to improve the efficacy of treatments compared to siRNAs or ASOs that can affect only a single target gene. Clinical trials of drugs based on miRNA-targeting ncRNAs have already begun, whether it is therapy to increase or decrease target miRNA, and they are being used in cancer treatment (50–52). Some miRNA markers in breast cancer are summarized in **Figure 2**. Although this strategy has shown great utility as an experimental tool, miRNA therapy has not yet been applied in the clinical treatment of breast cancer.

### 3.2 miRNA-targeted therapy in breast cancer

Circulating extracellular vesicle (EV)-derived miRNAs are now recognized as next-generation cancer “therapeutic diagnostic” tools with strong clinical relevance (53). Exosomes are involved in the transferring of miRNAs from donor cells to adjacent cells, acting as messengers between tumoral and stromal cells (54). Exosomes derived from cancer cells are not only enriched in miRNAs, but also contain a complete miRNA cargo, including pre-miRNAs, proteins involved in miRNA biogenesis and function, such as RISC loading complex (RLC), Dicer, trans-activating response element RNA-binding protein (TRBP), and AGO2, and thus pre-miRNAs can be processed to produce mature miRNAs (55, 56). Exosomes containing miRNA are taken up through receptor-ligand interaction, and subsequently regulate gene expression in recipient cells (57).

As one of the most prevalent and important post-translational modifications, ubiquitination is involved in multiple cancer-related pathways (58). Deubiquitinating enzymes (DUB) are involved in cancer regulatory processes by regulating ubiquitination. It was shown that miR-500a-5p was highly expressed in MDA-MB-231 and MCF7 cells treated with cancer-associated fibroblast (CAF)-derived exosomes. Upregulation of miR-500a-5p was also confirmed in CAF and CAF-derived exosomes. MiR-500a-5p is transferred from CAF to cancer cells and subsequently promotes proliferation and metastasis by binding to ubiquitin specific peptidase 28 (USP28). MiR-500a-5p promotes breast cancer progression and metastasis by sponging USP28 (59).

Yang et al. (60) found that exosomes produced by BC cells after stimulation with DOX or PTX delivered miR-378a-3p and miR-378d to neighboring cells to activate the WNT and NOTCH stemness pathways and induce resistance by targeting Dickkopf 3 (DKK3) and NUMB. In addition, chemotherapy activated the EZH2/STAT3 pathway in tumor cells, resulting in elevated levels of miR-378a-3p and miR-378d in cells and exosomes. More importantly, the combination of chemotherapeutic agents with the EZH2 inhibitor tazemetostat reversed chemotherapy-induced exosome-induced resistance in a tumor xenograft model in nude mice.

**Figure 3:**
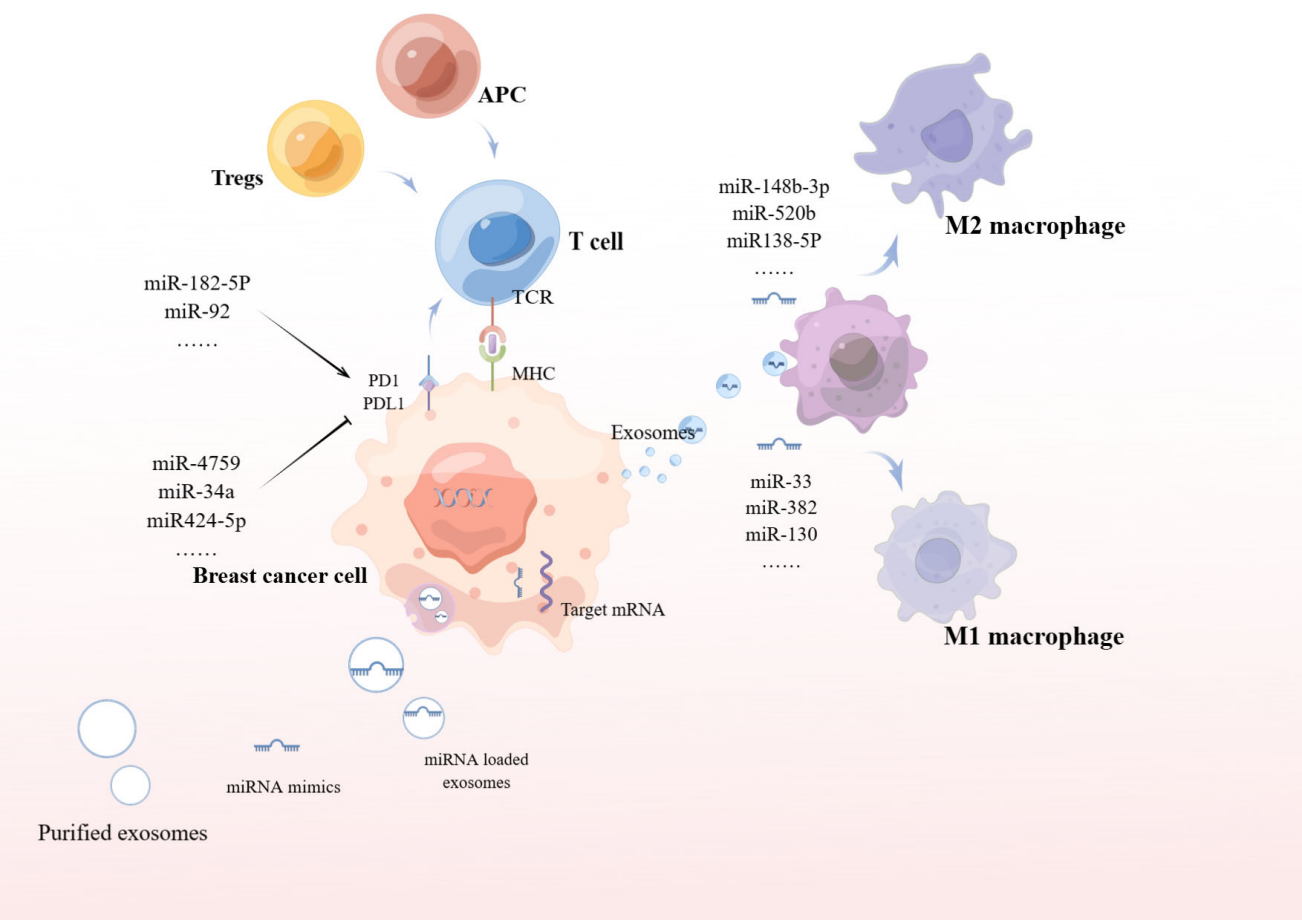
miRNA affects tumor cell immune function.

In addition to this, exosomes secreted by breast cancer cells deliver miR-148-3p, miR-520b, and miR-138-5p to target macrophages to induce M2 polarization, thereby promoting tumor growth (**Figure 3**). Thus, we can use exosomes to deliver antagonist tumor suppressor miRNAs for cancer therapy. In addition, removing exosomes from the circulatory system, or preventing the fusion/uptake of exosomes by target cells, can be used as therapeutic strategies to inhibit tumorigenesis. It can also be isolated from the patient’s circulatory system, modified, and relocated to the same patient for cancer treatment (61–63).

miRNA antagonists (antagomiRs) are synthetic oligonucleotides that target and antagonize oncogenic miRNAs of similar length. miRNA sponges are synthetic nucleotide structures that act similarly to antagomiRs in that they capture oncogenic miRNAs in the cell and impair their function. Transfection of metastasis-associated miRNA-10b overexpressing MDA-MB-231 cells with miRNA-10b-sponges resulted in decreased cell growth, migration, and invasion. MiRNA-10b overturning by miRNA-10b-sponges has been demonstrated to upregulate HOXD10, thereby inhibiting BC metastasis (64).

MiR-378 was downregulated in tamoxifen-resistant as well as chronically estrogen-deprived MCF7 cells (65). MiR-378 is growth inhibitory in ER-positive breast cancer and Arabkari et al. found that XBP1 (a transcription factor) was able to down-regulate the expression of miR-378 and PARGC1B (the host gene for miR-378) during UPR (a cellular stress response pathway involved in the maintenance of protein homeostasis in the endoplasmic reticulum). As a result their development of ORIN1001, an IRE1 inhibitor that blocks XBP1 production, is being evaluated for efficacy in a phase 1 trial in patients with advanced solid tumors or recurrent refractory metastatic breast cancer (66). A brief summary of potential therapeutic targets of miRNAs in breast cancer were shown in **Table 2**.

**Table 2:** Potential therapeutic targets of miRNAs in breast cancer.

miRNA	Cell	Target	Function	Reference
miR-500a-5p	CAF(exosome)、MDA-MB-231、MCF7	Acts as a molecular sponge for USP28	Promotes cell proliferation, migration, invasion, and EMT in breast cancer cells	(59)
miR-378a-3p 、miR-378d	BC cells stimulated with DOX or PTX	Regulates the WNT/β-catenin and Notch stemness pathways by targeting NUMB and DKK3	Induces chemoresistance	(60)
miR-206	MCF-7	Blocks the G1/S transition by targeting cyclin D2	Inhibites cell growth, migration and invasion and on down regulation causes apoptosis functions as a tumor suppressor	(71)
miR-200c-3p	MDA-MB-231、4T1	Inhibits the expression of *ZEB1* and *ZEB2*	Decreases tumor cell proliferation and induces cell cycle arrest in G2	(72)
miR-182	Py8119、TAM	Directly suppresses *TLR4*, leading to NFκb inactivation and M2 polarization of TAMs	Promotes macrophage alternative activation to drive tumor development	(73)
miR-378	MCF-7	Upregulates the type I interferon signalling pathway	Suppresses cell growth, colony formation, and migration of ER-positive breast cancer cells	(66)
miR-497/195	ER+ breast cancer cell	Reduced expression resulted in increased levels of miR‐497/195 target genes (AKT3, BCL2, MAP2K1, RAF1, and CCND1), which activated the of PI3K‐AKT signaling	Induces endocrine resistance and tamoxifen resistance	(112)
miR-142-3p	Different grade of breast cancer	High expression downregulates HMGA2 mRNA and protein levels inhibits the ERK/AKT/STAT3 signaling pathways	Induces apoptosis and G2/M cell cycle arrest in breast cancer cells and decreases cell proliferation	(113)
miR-18b	MDA-MB-231、MCF-7	Specifically bind to the 3’UTR of Transcription Elongation Factor A Like 7 (TCEAL7), and activate nuclear factor-kappa B (NF-κB)	Induces epithelial-mesenchymal transition (EMT) and promoting cell invasion and metastasis	(114)
miR-485-5p	MCF-7	Low expression upregulates MUC1 by binding to the 3’-UTR region	Promotes the proliferation, invasion and migration of breast cancer cells	(115)
miR-183	MCF-7、MDA-MB-231	High expression downregulates PTEN thereby regulating PI3K/AKT signaling path	Increases cell viability, accelerates cell cycle progression, and induces further migration of BC cell lines	(116)
miR-375	MDA-MB-231、MCF-7、MCF-7/ADR	Low expression upregulates JAK2 expression and its downstream effector P-STAT3 expression	MiR-375 stable overexpression via lentivirus infection reduces the stemness and decreases adriamycin resistance of breast cancer cells	(117)
miR-361-3p	MDA-MB-231、T47D	High expression decreases the expression of P73 by targeting E2F1	Promotes proliferation and inhibites apoptosis of breast cancer cells	(118)
miR-132-3p	MDA-MB-231、T47D	Low expression directly binds to the 3′UTR of the LAPTM4B gene and functiones at the post‐transcriptional level thereby regulating the PI3K‐AKT‐mTOR signaling pathway	MiR‐132‐3p significantly represses cell viability, colony formation, migration and invasion of breast cancer cells	(119)

### 3.3 Targeted delivery during miRNA therapy

Overexpression of some miRNAs that act as oncogenes in tumors may reduce the ability of tumor cells to undergo EMT, invasion and metastasis. However, delivery in cells remains the most important barrier to the use of miRNAs as therapeutic agents (67, 68). Significant down-regulation of miR-206 levels targeting NOTCH 3 has been reported in breast cancer cells compared to normal breast cells (69, 70). Chaudhari et al. (71) showed decreased expression of NOTCH 3 using up-regulation of miR-206 mimics by gold nanocomplexes, and miR-206 administered via gold nanocomplexes in MCF-7 cells was able to block cell proliferation, induce G0-G1 cell arrest, and alter mitochondrial membrane potential. Garrido et al. (72) used mesoporous silica nanoparticles to deliver miR-200c-3p for breast cancer therapy. MiR-200c-3p is a well-known tumor suppressor miRNA that inhibits tumor progression and metastasis in breast cancer by downregulating ZEB1 and ZEB2. They demonstrate that nanoparticles loaded with miR-200c-3p are a potential strategy for breast cancer therapy and a safe and effective system for tumor-targeted delivery of miRNAs.

In addition to delivering oncogenic miRNA factors, another study found that breast tumor cells induce miRNA (miR-182) expression in macrophages, and miR-182 promotes selective activation of macrophages to drive tumor development. Importantly, they found that loading miR-182 inhibitors using cationic mannan-modified extracellular vesicles and delivering the inhibitors specifically into macrophages effectively inhibited macrophage alternative activation and suppressed breast tumor development (73). Kardani et al. (74) inhibited miR-155 by designing a nanocarrier containing gold nanoparticles, antagomir-155, and a nuclear protein-specific aptamer. they reported a dramatic decrease in miR-155 mRNA levels and an increase in the levels of TP53INP1 mRNA, which is a direct target protein of miR-155.

Another promising delivery system to transport miRNA is the use of exosomes. The use of exosomes as delivery vectors for miRNAs may be effective in overcoming miRNA degradation *in vivo*, as exosomes can efficiently cross biological vectors and maintain communication with target cells. The biogenesis and targeting mechanisms of exosomes suggest that exosomes can optimize the expression of specific endogenous miRNAs and promote the regulation of multiple physiological mechanisms, including apoptosis in cancer cells (75). Nie et al. (76) found that once loaded with microRNA molecules in the exosome carriers, the resulting, miRNA-126 loaded 231-Exo (miRNA-231-Exo) strongly suppressed A549 lung cancer cell proliferation and migration through the interruption of the PTEN/PI3K/AKT signaling pathway. In addition, miRNA-126-loaded exosomes produced a potent lung homing effect in mice after intravenous administration of miRNA-126-loaded exosomes.

## 4 Potential therapies for other non-coding RNAs in breast cancer

CircRNAs act as miRNA sponges to regulate endocrine resistance (77, 78). Xia et al. (79) found that miR-217 expression was reduced, while G3BP2 was overexpressed in BC tissues. G3BP2 was verified as a direct target of miR-217 by luciferase assay. Inhibition of G3BP2 expression inhibits cell migration of BC cells. Paclitaxel-induced exosome circBACH1 regulates BC cell stemness and migration by sponging miR-217 to upregulate G3BP expression, which provides a new therapeutic target for paclitaxel resistance and BC progression through the circBACH1/miR-217/G3BP2 axis. Multiple studies have demonstrated that miR-204-5p is down-regulated in breast cancer patients and MCF-7 cells (80–82). Jiang et al. (83) demonstrated that circRHOT1 acts as a sponge for miR-204-5p and promotes breast cancer cell invasion and epithelial-mesenchymal transition (EMT). They found that miR-204-5p targets the protein arginine methyltransferase 5 (PRMT5) and shows an opposite expression pattern, and thus reversed EMT by overexpressing PRMT5 to reverse the effects of circRHOT1 knockdown on cell growth, apoptosis, wound healing, and cell invasion, as well as on the expression of E-calcineurin, N-calcineurin, and poikilodulin.

SiRNA is an RNAi tool with the ability to inhibit target genes. Li et al. (84) developed an endosomal pH-responsive nanoparticle that carried Rac1 siRNA along with cisplatin, which resulted in efficient delivery of Rac1 targeting oligonucleotides and cisplatin in breast tumors and showed promising synergistic antitumor effects. Wu et al. (85) used lipid-coated calcium phosphate nanoparticles to inhibit PD-1 and PD-L1. This allows the siRNA to efficiently enter the MCF-7 BC cell line and subsequently inhibit the PD 1 receptor and ligand.

The clustered regulated interspaced short palindromic repeats (CRISPR)/Cas9 system is emerging as a powerful tool for precision medicine as a revolutionary and viable genome editing tool (86–88). Mao et al. (89) targeted EZH2 with the CRISPR/Cas9 system and inhibited EZH2 mRNA and protein expression in MDA-MB-231 cells, whereas knockdown of EZH2 inhibited the proliferation and migration of MDA-MB-231 *in vitro*. Based on the role of CRISPR/Cas9, many experts considered that some nanoparticles could be designed for efficient targeted delivery of CRISPR/Cas9 plasmids (90–92).

## 5 Discussion of breast cancer therapies

Traditional treatments for breast cancer mainly include surgical excision, radiotherapy, chemotherapy, endocrine therapy, targeted therapy and immunotherapy. Among the available treatments for HER2-positive breast cancer, the combination of trastuzumab, patuximab and paclitaxel analogs (THPs) is still the preferred first-line treatment (93). HR-positive/HER2-negative breast cancer is treated primarily with hormone therapy, and intermediate- and high-risk patients may receive concurrent chemotherapy (94). First-line standard therapy for metastatic patients is CDK4/6 inhibitors in combination with hormone therapy (95). Treatment for triple-negative breast cancer then includes the PD-L1 inhibitor Tecentriq and the PD-1 inhibitor Keytruda, as well as the PARP inhibitors Lynparza and Talzenna (96–98). These methods play an important role in the treatment of breast cancer, but have some limitations. For example, although chemotherapy can kill cancer cells, it may also harm normal cells and bring about side effects; whereas endocrine therapy and targeted therapy require the patient’s tumor to have the corresponding receptor expression or gene mutation in order to be effective. ncRNA therapy has a number of potential advantages over conventional treatment. First, ncRNA therapies may be more precise because they can target specific molecular pathways or signaling networks, reducing the impact on normal cells. Second, ncRNA therapies may help overcome the problem of drug resistance to conventional treatments because they can intervene in the biological behavior of tumor cells from a new perspective. In addition, ncRNA therapies may have a better safety profile and fewer side effects because they are based on modulating endogenous molecules rather than introducing foreign chemotherapeutic agents (45). The ncRNA-based diagnostic field has much advanced with numerous diagnostic tools already offered to clinical trials (**Table 3**).

**Table 3:** NcRNA therapies already in clinical trials.

NcRNA Therapies	Cancer	Clinical Trial Number	Reference
siRNA	BC	NCT06357689	(120)
ASO	BC	NCT01563302	(121)
shRNA	Sickle Cell Disease	NCT03282656	(122)
siRNA nanoparticle	Glioblastoma	NCT03020017	(123)
miRNA liposomal formulation mimic	HCC、 Colorectal、 Leiomyosarcoma	NCT01829971	(50)
EnGeneIC Dream Vectors、 mimic microRNA	Malignant pleural mesothelioma	NCT02369198	(52)
Miniature biodegradable polymeric matrix loaded siRNA	Pancreatic ductal adenocarcinoma	NCT01188785	(124)
Exosome delivery vehicles with siRNA	Pancreatic cancer	NCT03608631	(125)
LNA Selective inhibitor	Refractory advanced cancer	NCT04811898	(126)

The clinical application of ncRNAs as potential therapeutic targets for cancer can be manifested in two ways: the use of ncRNAs to “complement” inhibited or missing RNAs (replacement therapy) or to “block” the action of overactive oncogenic RNAs (99). However, the existence of many different ncRNAs associated with BC suggests that the regulation of BC is more complex than we expected. In this aspect, we need to further investigate the possibility that the expression of ncRNAs changes during the course of the disease, just as other oncogenic molecules do in cancer. Therefore, specific targeting of different ncRNAs may be required to effectively combat disease recurrence. In addition, targeting multiple ncRNAs can be rationally supplemented for effective BC inhibition. Determining which ncRNAs to target may depend on the specific expression profile of each patient to realize the idea of personalized medicine (100). Another aspect to consider is the complex interactions between different ncRNAs. As in breast cancer the anti-tumor miR-149-5p is sponged by both CircFAM64A (101) and Circ_0072995 (102) molecules, implying that CircFAM64A and Circ_0072995 may need to be silenced simultaneously. Also, it has been shown that just one miR cluster (miR-15a-16-1 cluster) can affect (through direct and indirect targeting) approximately 14% of the entire transcriptome in leukemia cells (103). Finally, no siRNAs targeting lncRNAs are currently in clinical trials due to a variety of challenges, including “off-target” issues, inefficient delivery to tissues and cells, and non-specific immune responses. “Off-target” issues can lead to unexpected transcription and protein silencing, false-positive hits and cell growth inhibition, as well as competition with endogenous non-coding RNAs. siRNAs are inefficiently delivered primarily due to their tendency to accumulate and be absorbed predominantly by the liver, and the challenge of delivering siRNAs to other tissues remains. Therefore, optimizing anti-ncRNAs therapeutics must also take into account the delivery of therapeutic molecules.

## 6 Conclusions

BC is one of the most common diseases globally, and its incidence continues to rise despite long-term efforts to reduce its impact on human life. Conventional BC therapy remains inadequate due to the heterogeneity and high chemoresistance of this disease. Currently, most of the studies on ncRNAs regulating tumorigenesis and development are mainly cellular or animal experiments, and clinical research is still in the initial stage, and more clinical trials need to be carried out in the future to find out more safe and effective ncRNAs with universal or tissue specificity and specific ncRNAs for different tumor types. While advances have been made with non-viral delivery systems such as lipid nanoparticles (LNPs), further improvements in their targeting and reduction of immune responses are still necessary. NcRNAs are diverse and functionally distinct, and the complexity of their mechanisms of action in cells adds to the difficulty of understanding their role in disease. NcRNA therapies may also need to be personalized to an individual’s genome and disease characteristics. However, sufficient technological advances have been made to synthesize and manufacture most of the ncRNA mimics and inhibitors for use in preclinical studies and eventually in human clinical trials. In conclusion, although the field seems to be in its infancy, we are currently witnessing the growing potential of ncRNAs in cancer therapy. There is a need to further elucidate the development and clinical application of ncRNAs in breast cancer research to provide a theoretical basis for biomarkers and targeted therapies for breast cancer.
